# Cervical Cancer Screening Uptake and Experiences of Black African Immigrant Women in Canada

**DOI:** 10.1177/23333936241266997

**Published:** 2024-07-27

**Authors:** Abosede C. Ojerinde, Sally E. Thorne, A. Fuchsia Howard, Arminee Kazanjian

**Affiliations:** 1 University of British Columbia, Vancouver, Canada

**Keywords:** Black African immigrant women, Canada, cervical screening, interpretive description, socioecology

## Abstract

Cervical cancer is one of the leading causes of cancer-related death among African women. Unfortunately, in most sub-Saharan African nations, women are vulnerable if they are unaware that cervical cancer is preventable with frequent screening and early treatment. The aim of this study was to examine Black African immigrant women’s perceptions and experiences of cervical screening in British Columbia, Canada. Twenty Black African immigrant women were interviewed using the qualitative research method Interpretive Description. Data collection approaches included indepth interviews and analytic memos. Data were analyzed using a constant comparative technique guided by a socioecologic framework to capture subjective experiences and perceptions. Four key themes were identified, including confusing conceptualizations about cancer and cancer screening, competing priorities, concerns for modesty, and commitment to culture. The study findings point to the need for more active approaches to promoting cervical screening for this population.

## Introduction

Worldwide, cervical cancer is the eighth most frequently occurring cancer overall, ranking fourth for incidence and mortality (Bray et al., 2018). In Canada, cervical cancer is the 13th most diagnosed cancer among women and the incidence and mortality have decreased considerably over the past few decades, primarily because of coordinated screening and early detection systems (Canadian Cancer Society, 2017). Cervical screening is a primary screening tool used to identify asymptomatic individuals with abnormal cell changes in the cervix that may indicate an increased risk of developing either cervical cancer or a precursor of the disease. The Pap smear test is performed to detect the presence of preinvasive or cancerous cells in the cervix, and women are recommended to partake in regular screening to detect cell changes before they become cancerous or while treatment can be effective. The Canadian Task Force on Preventive Healthcare (2018) has developed national guidelines for cervical cancer screening, which currently recommend routine screenings every 3 years for women aged 25 to 69 years.

Despite the widespread availability of publicly funded cervical screening programs in Canada, participation rates among immigrant women, as well as those from ethnocultural minorities, remain poor in most provinces (Bacal et al., 2019; Canadian Partnership Against Cancer, 2022; Ferdous et al., 2018). As the literature confirms, no single factor serves as a primary barrier to cervical screening for all immigrant or ethnocultural minority women, given their varying histories, circumstances, and interactions with healthcare services, thus making this a complex phenomenon to understand. Qualitative studies conducted on the cervical screening practices of Canada’s highly diverse immigrant population have tended to focus on immigrants from a specific country of origin; for example, studies conducted among women of Asian, Korean, Chinese, and Arab descent suggest that barriers to screening include women’s limited knowledge about screening and cervical cancer, language barriers, transportation, childcare, low socioeconomic status, preference for a female physician, fatalistic beliefs, and modesty (Cha & Chun, 2021; Hulme et al., 2016; Redwood-Campbell et al., 2011).

Canada’s ethnocultural immigrant communities are linguistically, culturally, and spiritually diverse (Statistics Canada, 2018). Studies conducted on immigrant women from sub-Saharan Africa living in the United States, United Kingdom, and Australia show that their screening uptake is influenced by several characteristics of culture, structural forces, and socioeconomic factors, and that poor screening uptake may be partly attributable to such factors as fear of stigma and embarrassment associated with women circumcision and fatalistic beliefs (Adegboyega & Hatcher, 2017; Anaman-Torgbor et al., 2017; Ghebre et al., 2015; Huhmann, 2020). Although some authors have investigated the perspectives of immigrant women and healthcare providers in Canada, and the cervical cancer experience of Black women in a more general sense has received some research attention (Christy et al., 2021), the viewpoints of Black African immigrant women in the Canadian context have remained relatively unexplored (Cénat et al., 2023). Thus, little is known about how their screening experiences are affected by what is a recognized diversity in their cultural heritage or social context (Christy et al., 2021). There is also limited understanding of how broader systems and policies related to settlement and healthcare may shape Black African immigrant women’s cervical screening uptake and their healthcare utilization in general (Cénat et al., 2023).

## Problem, Purpose, and Research Questions

Organized population-based cervical screening programs are not available in most Sub-Saharan African countries (Johnson et al., 2018; Pierz et al., 2020). According to Yimer et al. (2021), in 2017, cervical cancer screening coverage was 10% in sub-Saharan Africa and, in the four West African countries they studied, less than 1% of women had ever been screened. Cervical cancer is the second leading cause of women cancer mortality in this region, and about 80% of women diagnosed die (Okunowo et al., 2018; WHO, 2023). In Canada, cervical cancer incidence and mortality have decreased for several decades because of the early detection and treatment of the precursor lesion in the organized provincial cervical screening programs (Caird et al., 2022; Canadian Partnership Against Cancer, 2022). Evidence shows that, despite widely available screening programs, cervical cancer is less likely to be detected early in immigrant women than in the general population because immigrant women tend not to take advantage of the screening services (Health Canada, 2019).

In a recent scoping review of factors affecting cancer rates among Black people in Canada, furthermore, Cénat et al. (2023) concluded that the lowest-income group among sub-Saharan African immigrant women is likely at highest risk for not undergoing cervical cancer screening. With this understanding, it is important to investigate women’s experiences using these services as well as their reasons for non-utilization of cervical screening. Thus, the purpose of this study was to explore this within the broader context of the everyday lives and general experiences with healthcare for Black African immigrant women in Canada, including any structural and systemic problems that might constitute barriers to cervical screening uptake. The following research questions guided this study: (a) What barriers related to the different contexts of the Canadian healthcare system and society influence Black African immigrant women’s cervical cancer screening uptake? (b) How do Black African immigrant women in Canada conceptualize cervical screening, and how does their conceptualization influence the use or non-utilization of cervical cancer screening?

## Methods

### Design: Interpretive Description

The study employed a qualitative research design based on an Interpretive Description (ID) approach to applied and practice inquiries (Thorne, 2016). ID studies recognize that social reality is shaped by human experiences and social contexts (Thorne, 2016). Cervical screening uptake can be understood as a socially constructed experience, specifically regarding the implications of such complex and intersecting dimensions as gender and culture. An ID study produces knowledge that is useful to extending the applied knowledge available to practice disciplines such as nursing (Thorne, 2016).

### Theoretical Framework

The theoretical fore-structure guiding this study design and implementation drew upon the socioecological theoretical framework (Stokols, 2018), applied with a nursing disciplinary orientation. The socioecological theoretical framework was chosen because of its emphasis on understanding the dynamic interrelations between various individual and environmental barriers to cervical screening. Grounding this research within the applied perspective of ID methodology from a nursing perspective meant that we acknowledged that multiple realities exist and will influence the meaning making process related to women’s cervical screening experiences. Therefore, in keeping with this perspective, the Black African immigrant women’s cervical screening experiences were explored in a way that allowed for the description of multiple and intersecting realities in these women’s lives. Given that nursing is committed to understanding the contextual and lived experiences of the client in interaction with their environment, ID’s recognition of the socially constructed nature of human experience (Thorne, 2016) aligns with how we view nursing practice. As such, grounding the study in these theoretical perspectives shaped the interpretation of the data and provided understanding of the relationship between an individual and their social environment with regards to their health (Kilanowski, 2017). Further detail about the theory and methodology guiding this study is available in Ojerinde (2023).

### Sample and Setting

Black African immigrant women were purposively sampled from around the Greater Vancouver Regional District, the largest urban area in the province of British Columbia (BC), Canada. We used multiple recruitment techniques such as fliers and posters, consultations with community leaders, and snowball sampling. The inclusion criteria were Black African immigrant women who had immigrated from any one of the 48 sub-Saharan African countries; self-identified as Black African; were 25 to 69 years of age (which is the age of eligibility for cervical screening in BC); and spoke and understood English. We were open to the participation of women regardless of whether they were a Canadian citizen, Permanent Resident, or recent immigrant, irrespective of years of residence in Canada. The exclusion criteria were women with a history of a cervical cancer diagnosis and/or a total hysterectomy. Recruitment occurred from May 2020 to March 2021. The demographic characteristics of the sample are shown in Table 1.

**Table.1.** Participant Demographic Characteristics (*n* = 20).

No.	Age	Marital status	Country of origin	Immigration status	Years of residency	Education	Employment status	No of children
1	39	Single	Nigeria	Citizen	24	Graduate degree	Employed	0
2	47	Married	Zimbabwe	Citizen	28	College diploma	Employed	1
3	28	Single	Nigeria	Permanent resident	2	Graduate degree Degree	Employed	0
4	45	Married	Nigeria	Recent immigrant	1	Graduate degree Degree	Unemployed	2
5	39	Married	Nigeria	Permanent resident	5	University degree Degree	Employed	3
6	48	Married	Malawi	Citizen	12	College diploma	Employed	3
7	42	Married	South Sudan	Citizen	15	College diploma	Unemployed	4
8	55	Married	Swaziland	Permanent resident	10	University degree	Employed	2
9	32	Cohabiting	Benin	Permanent resident	2	Graduate degree	Employed	0
10	35	Single	Rwanda	Recent immigrant	6	Graduate degree	Employed and schooling	0
11	39	Married	Zimbabwe	Citizen	7	University degree	Employed	2
12	39	Single	Nigeria	Permanent resident	2	University degree	Employed	0
13	50	Married	Zimbabwe	Recent immigrant	3	Graduate degree	Unemployed	4
14	35	Married	Ghana	Permanent resident	3	Graduate degree	Employed	1
15	45	Married	Zimbabwe	Citizen	17	College diploma	Employed	2
16	31	Single	Nigeria	Permanent resident	10	University degree	Employed	0
17	25	Single	Zimbabwe	Recent immigrant	3	Graduate degree	Unemployed Schooling	0
18	53	Married	Nigeria	Recent immigrant	2	University degree	Employed	3
19	44	Married	Botswana	Recent immigrant	3	Graduate degree	Employed	2
20	46	Married	Zimbabwe	Citizen	22	University degree	Employed	2

*Note*. All participants had high school completion as well as a college diploma or university degree, some also had graduate level university education.

### Ethics

The study received approval from the Behavioural Research Ethics Board of University of British Columbia Behavioral Health Research Board Project # H20-00611 and followed the guidelines stipulated by the Canadian Tri-Council Statement. Informed written and/or verbal consent was obtained from all study participants with the understanding that there was no direct benefit to participants from taking part in this study.

### Data Collection

Data collection with the 20 women in this study was conducted through indepth virtual interviews. Although both telephone and the Zoom interview were offered as options, only one participant opted for the latter. Interviewing was done by the first author, a nurse who is herself a Black African immigrant woman (from Nigeria). Data obtained from the interviews, which ranged from 60 to 90 min in length, were audio-recorded and subsequently transcribed, and were augmented by the creation of analytic memos and a reflexive journal, including reflections on the extent to which the interviewer’s positionality may have facilitated and/or complicated aspects of the data obtained. Given the complexity of their lives, interviews took place in a location most convenient to the women: at their home, in their office, or in instance, while driving. The same interview guide (Table 2) was used to structure all interviews, but each participant’s interview evolved uniquely, probing for additional detail, and responding to cues from individual perspectives and personal experiences. The open-ended approach was designed to engage participants in conversations that would enable gathering individualized information to enhance a comprehensive understanding of each individual case, while also ensuring common data across the sample.

**Table 2.** Interview Guide.

1. Can you tell me a little bit about your life in Africa and your life since coming to Canada? 2. Now that you have spent some time in Canada, what is your everyday life like these days? 3. How do you manage your family and your work? Was this what you expected? 4. Can you tell me the overall experience you have had in getting healthcare in Canada? 5. Can you describe some of the specific experiences you have had using healthcare in Canada? 6. Is there a moment or memory that stands out for you? 7. Tell me what you understand about staying healthy and prevention of diseases. What do you know about women’s health and cancer prevention? 8. What do you feel about cervical cancer screening? 9. Have you had a Pap smear test before? 10. If yes, tell me about your experience last time you had the test? 11. Can you describe how you felt when you had the test? 12. What would you like to have happened? 13. Can you share your major concerns about the Pap test? 14. How did you address such concern? How did your doctor address the concerns? 15. Will you continue to have a regular Pap smear test? If so, why, if not, why? 16. If no, what do you know about cervical cancer? 17. What is your understanding of why Pap tests are recommended for all women of your age? 18. What kinds of feelings do you have when you think about going for a Pap test?

### Data Analysis

Thorne et al. (2004) recommend concurrent data collection and analysis, such that each process informs the other iteratively. Our process involved constant comparative, inductive and interpretive techniques between and among the interview data, analytic memos, and reflexive journal material. We transcribed and reflected on each interview before performing the next, such that preliminary data exploration and inductive analysis commenced as soon as the first interview was completed. Through continuous reflection on the evolving data set and our desire to seek out heterogeneity within the sample, we continually sought to maximize the likelihood of obtaining sufficiently rich and diverse data to address the research questions through the strategy of seeking out participants representing a range of demographic characteristics and experiences with cervical cancer screening (DiCicco-Bloom & Crabtree, 2006). In this manner, in considering each interview transcript, and in seeking to recruit additional participants, we drew on the socioecological theoretical framework to reveal structural and individual conditions associated with the participants’ cervical screening experiences.

The concept of “information power” (Malterud et al., 2016, p. 2) helped guide our determination of when to stop data collection. Information power refers to the qualities and features of data acquired rather than quantity. Therefore, we gathered data until we obtained sufficient richness, from an appropriate sample including the participants’ extensive knowledge of the phenomenon under study, to generate meaningful findings. We managed data using word-processing files and functions, rather than available software systems, primarily to avoid premature coding and allow us to consider multiple potential organization strategies for display of findings before deciding on the optimal approach to our findings report. Data analysis and management were done by the first author with the active support of all coauthors.

## Findings

To contextualize the thematic findings that resulted from our analytic process, it is important to situate the participant sample within the larger population of Black African immigrant women in this region. Although many of the study’s participants recognized the benefits of engaging in BC’s cervical cancer screening program, some found it difficult to follow the recommendations. When asked if they had ever had cervical screening, 17 indicated they had. Nine of the 17 had been screened in their countries of origin, or other Western countries, as well as in Canada; 6 had been screened in Canada; and 2 women had only had screening in their home countries. The remaining three women stated that they had never been screened. Thus, in each interview, we attempted to situate each participants’ access to cervical cancer screening within her own settlement experiences and the larger contexts of her life as they had been shaped by the gendered familial expectations that persisted following their arrival in Canada. Our findings are therefore organized by describing the complex nature of this screening uptake context before we present the thematic findings as a set of conceptualizations within which their screening decisions and actions become understandable.

## The Context of Black African Immigrant Women’s Cervical Screening Uptake

Since the women’s experiences with migration and settlement, their entry into Canadian society and their involvement with the healthcare system intersected in different ways with their experiences with cervical screening uptake, it is necessary to set the stage by describing their lives in some context. Many of their accounts revealed that one unpleasant situation led to another as they attempted to interact with BC’s social systems and government bureaucracy and find their place in Canadian society. Their challenges included keeping up with traditional gender roles which included performing paid work to contribute to household finances, performing domestic tasks and childcare. They played these roles while dealing with challenges related to social isolation, a lack of English language proficiency, financial instability, issues relating to their social identities, and perceived discrimination on both the personal and structural levels. Throughout these challenges, however, it seemed that the idea of maintaining some traditional African gender norms was a source of personal strength aligned with their self-identity.

Accessibility to health care was also an important determinant of their understanding of what might be required for them to participate in the BC’s cervical screening program, and the decisions they could make about cervical screening. All the participants were registered with the provincial health insurance plan and eligible for publicly funded medical care. All recent residents, except for two, and two long-term residents (i.e., 10 out of 20) reported that a central concern was the difficulty in finding a primary care provider for reliable care and information on primary health concerns. An inability to secure a regular family physician or nurse practitioner was a significant impediment to their effective utilization of healthcare resources. Effective utilizing of the healthcare system entails an individual’s ability to obtain information and services when needed and to interact with healthcare providers to make informed choices about care needs (Liu et al., 2020). However, all the participants reported that they had faced a variety of complex and problematic issues in using BC’s healthcare system. Overall, and in varying ways, the women’s cultural values, traditional gendered roles, migration, and settlement experiences appeared to have shaped the broader social and structural contexts of their daily lives, within which they encountered systemic barriers to obtaining healthcare services and, possibly, cervical cancer screening.

## Participants’ Conceptualizations of Cervical Screening

We present our findings pertaining to these women’s conceptualization of cervical screening according to four common themes: (a) confusing conceptualizations, (b) competing priorities, (c) concerns for modesty, and (d) commitment to culture. Within these themes, we describe how these conceptualizations may have shaped how each individual woman perceived, interpreted, negotiated, and understood cervical cancer screening uptake in the context of her personal circumstances and experiences. In addition, we employed the lens of the socioecological perspective that guided the study’s process to try to identify a complex set of interrelated forces that were relevant in the context of these African immigrant women in Canada. From this perspective, we were able to categorize the barriers to cervical screening for these women as operating across and within the various levels of the socioecological theoretical framework, including factors at the individual, interpersonal, organizational, community, and system levels (McLeroy et al., 1988; Stokols, 1996).

### Confusing Conceptualizations of Cancer and Cervical Screening

Confusing conceptualizations about the nature of cancer in general and cervical cancer in particular were dominant in many of the women’s narratives. Although all the participants identified as Christians, several acknowledged their view that Black Africans in Canada still hold indigenous beliefs in tandem with their Christian beliefs, and that these will have shaped their values, attitudes, perspectives, and decision-making paradigms regarding healthcare and cervical screening uptake. When asked about their general understanding of cancer, some participants provided descriptions of cancer that seemed to blend various biomedical, religious, and cultural perspectives. For example, one woman, who had never been screened, explained it this way:

Concerning cancer, I know it’s normally present with some type of growth or something, and its. . .uhm. . .one of those things that once it starts developing, you see it and when you visit a doctor, they can tell that it's cancer. But, in my country, I don’t think they even call this disease cancer. To them, it appears to be a bewitching performance by someone, at least that is what they believe.

Similarly, several participants provided conceptualizations of cervical cancer that directly associated the disease with death. As one woman described it: “When a woman develops cervical cancer, she dies.” Another depicted it this way: “Cervical cancer is a type of cancer that grows on or affects the womb’s neck, and, like any other cancer, it kills.” Regarding the purpose of cervical cancer screening, all but two women had only a vague idea about the primary purpose of cervical screening. Of those who did believe they understood it, the explanations still held elements of uncertainty:

Checking your insides to see if there is anything abnormal that could lead to cancer for the proactive measure of checking the place, what’s happening, what’s going on. To detect the growth of abnormal cells, which could lead to cancer. That’s what I think it is, but I could be wrong.

In addition, some of the participants’ understandings of screening reflected confusion about the recommended frequency of screening in the BC cervical screening program. One explained: “I get my Pap smear test done every year because I’m over 50. But it is now every two years,” while another reported: “I did it every two years before but it’s now every year.” Yet another woman misinterpreted the screening frequency because she had associated the period around childbirth with cervical screening schedules. “When I went back to my midwife when I was going to have my daughter, which was my last child, she told me that it was about to expire, so I need to do it again. I think it’s every three years or something like that.” These responses suggested that, even though these women may have had screening both outside of Canada and in BC, they may have either forgotten the recommended frequency or became confused between the recommendations in the various parts of the world where they had had access to screening.

Confusing conceptualization may have an impact on how an individual interacts with others on an interpersonal, organizational, and communal level and may either predispose or enable a woman to use cervical screening services. As previously stated, all the participants identified as Christians, and as such, the church, for example, may recommend fasting and prayer to protect people from developing diseases, including cancer. Such practices may reinforce a woman’s beliefs about the role of supernatural forces in cancer, and create the impression that only God can protect them from these powers. As a result, in the absence of adequate screening information or clear screening recommendations, some of these women may continue to pray and fast, believing that their faith will protect them from developing diseases.

### Competing Priorities

The participants’ accounts repeatedly emphasized the importance of considering the many interconnected forces that produced the social context in which they lived in BC. The married women suggested that the multiple competing priorities, including family pressure and paid job, made it difficult to consider cervical screening an immediate priority. It was evident from the interviews that ingrained cultural beliefs and commitment to social expectations in the community may also have influenced some of these women’s cervical screening practices. One woman pressured by lack of time explained her busy life this way:

More than anything, family pressure. You know what, I guess we get busy. That’s just the way life is here. As an example, in the last year or two, in addition to working as a nurse, I also worked as a clinical instructor on-site for a private college. My children are also involved in sports. So, while I’m not at work, I feel that those four or five days off belong to them. I don’t necessarily prioritize myself; I think I prioritize the children more than I do myself.

Another woman who seemed preoccupied with resettlement issues said:

You can’t start talking about healthcare if there’s nothing wrong with you. As a result, you cannot begin to consider how to obtain healthcare services because it will not be on your mind. You would concentrate on how to get work, support the family, and settle down. So, I have been thinking. . .. How do I get into the healthcare here to have my body checked and all that? So, once I settled down, I intend to learn more about cervical screening.

Thus, in keeping with the socioecological theoretical perspective, we identified a complex set of multiple interrelated conflicting priorities. These priorities within their social environments also served to create barriers at different socioecological levels. For instance, at the individual, the interpersonal and the community levels, work time constraint in the absence of symptoms combined with women’s tendency to prioritize family above themselves because of social expectations interrelated with structural forces such as the lack of an explicit screening recommendation and difficulty navigating the healthcare system to create barriers to screening adherence.

### Concerns for Modesty

All participants spoke of the value of modesty, including social constraints regarding being touched by a stranger, preferences around the gender and status of the physician, and religious and cultural beliefs; in part, this value made adhering to screening recommendations challenging. Regarding concerns for modesty, when asked how they felt about their most recent cervical screening, a few were reluctant to elaborate, while others provided specific details. Several participants’ comments suggested that the gender of the healthcare provider was important to them with respect to cervical screening. One woman shared a general preference in this regard: If I have an option between a man and a woman, I will choose the woman. I believe it is more comfortable for me to be naked in front of a woman doctor. A few of the women explained that their strong preference to have cervical screening done by a woman gynaecologist made it challenging for them to receive a referral in BC. One explained:

When I arrived here, I discovered that I needed a referral to see a female gynaecologist. So, I got a referral to the woman’s home, and that one referred me to another, and I finally got a gynaecologist. The gynaecologist then directed me to another location, where I was scheduled for an appointment. The wait for a female gynaecologist appointment in Vancouver was almost a year. That was mind-blowing (laugh).

However, for other women, provider gender was clearly a strong deterrent. As one shared: “Indeed. I have never had a male gynaecologist and have no plans to do so. I’d never considered having a male gynaecologist.” I don’t mind going for cervical screening if I can find a woman gynaecologist, which is my first criterion. As a related issue, comments made by some participants suggested prior undesirable experiences which seemed to have acted as barriers to screening adherence, ultimately increasing their risk. In describing their previous screening experiences, some of the women used expressions such as very uncomfortable, extremely uncomfortable, painful, and it’s scary.

From a socioecological perspective, personal characteristics could contribute to women’s reluctance to undergo cervical cancer screening because it appeared that many of the women considered the gender of the family physician or gynaecologist an important factor in considering whether to be screened. Several of the women’s comments about sensitivity to the cervical screening process and the undesirable experiences they had encountered previously may have interrelated with structural forces (e.g., gender of the provider, time constraint), becoming barriers to screening adherence at both individual and organizational levels, and ultimately increasing their risk.

### Commitment to Culture

Considering the participant’s comments concerning sensitivity to the cervical screening procedure, it also became apparent that some of the women may hold culturally related social norms related to sexual behavior and femininity, including taboos around discussing issues related to women anatomy. To comprehend how culture had informed their attitudes on sexuality and how this in turn had influenced their thoughts on screening uptake, we asked them about any experiences they might have to talking about women’s genitalia and similar subjects.

All participants shared that discussing women genitalia is considered taboo in their African culture, and any open discussions of such issues constitute a violation of cultural norms. This raises the interesting contradiction that, even if they felt free to participate in cervical cancer screening, the subject should not be discussed. This was apparent in the interviews, where almost all the women (18 of the 20) seemed quite uncomfortable mentioning the cervix or vagina by name, avoiding the use of the exact terms for female genitalia when describing the cervical screening procedure. Several used euphemisms such as down below, the under, the area, the inside, and down there, when referring to women’s genitalia. Some women elaborated on their responses and shared that what they learned while growing up may have influenced their attitudes toward discussing genitals and sexuality. For example, one shared this perspective:

I realized recently that my daughter calls her genitals bum-bum. She was touching it saying, “I’m touching my bum-bum.” She has not even figured out that they are two different things. We’ve been so confused. She is aware of her eyes, ears, nose, and mouth, but she doesn’t know the vagina because me and my husband avoid using the word. We have been going back and forth and trying to figure out what name to call them (laugh). I’m thinking JJ. . .. Or should we call it coocoo? (laugh).

She went on to explain further:

I remembered that my mother gave the penis a name. So, I called my mother and asked, “What did you call the vagina when we were kids?” She claimed she couldn’t recall the name. You know, the fact that we can't refer to it is where the problem starts, and she [her daughter] is only three years old. It still makes me very, very uncomfortable (laugh).

In a similar manner, being unmarried may also intersect with a lack of ongoing access to a trusted primary care provider to reinforce poor knowledge of what constitutes personal risk. Therefore, factors affecting the women at the individual level cannot be separated from the interpersonal level because society’s existing social context and structural forces intersect with individual social identities. Thus, it was evident in these women’s accounts that a persisting commitment to their traditional culture, including norms around fulfilling their gender role as a woman and of modesty, intersected with the complexities of their lives as immigrants in Canada to render cervical cancer screening, and indeed to their health maintenance generally, as a lesser priority.

## Discussion

In considering the study findings, we reflect on them in the context of theoretical perspectives found in the literature on cervical cancer screening and examine what the use of the socioecological theoretical framework lens can add to this initial consideration to provide a more indepth understanding of the uptake of cervical cancer screening by Black African immigrant women in BC. In that it recognizes multiple levels of influence and supports the idea that behaviors affect, and are affected by, various contexts within the environment (McLeroy et al., 1988) the socioecological framework allows for the possibility of considering active approaches to address the problem of cervical screening uptake within the community of Black African immigrant women, and also potentially for Black African immigrant women in other Canadian regions and within other similarly challenged immigrant populations and subgroups in the Canadian context, in ways that draw on forces at play at the individual, interpersonal, institutional, communal, and societal levels.

Previous studies have also drawn on the socioecological perspective to examine barriers to cervical screening and mammography among different immigrant women in Europe and North America (Ali et al., 2021; Cha & Chun, 2021) and among Somali immigrant women in Norway and in the United States (Gele et al., 2017; Ghebre et al., 2015). In this study, the participants’ personal characteristics, including their knowledge, attitudes, behaviors, marital status, immigration status, language barriers, and family backgrounds were considered as influential individual level forces. These characteristics can either predispose or enable individuals to utilize screening services or, conversely, may serve as barriers to accessing such services. These individual level factors can also be influenced by factors in all other levels of the socioecological framework (McLeroy et al., 1988). For example, as was apparent in this study, factors associated with the individual level are often the product of interpersonal interactions and broader sociocultural influences. These same kinds of interactions that have been reported among others who have studied sociocultural and structural forces shaping the uptake of cervical screening among African immigrant women (Addawe et al., 2018; Adegboyega & Hatcher, 2017; Ghebre et al., 2015; Idehen et al,. 2020), and our findings expand upon them to illustrate how these interactions played out in a diversity of individual cases. At the systems level, they demonstrate the critical importance of the human need for a safe environment (Carayon et al., 2014), and illustrate the many mechanisms at play that could render the health care system sufficiently unsafe for these women that their access to recommended services was impaired. The study findings suggest that a healthcare system that embraces innovative approaches to outreach—approaches that are informed by consideration of all levels of the socioecological framework and more actively engage women in cervical cancer screening—is warranted.

### Implications for Practice

As previously stated, the absence of adequate screening information accessible to the community and the lack of explicit recommendation toward screening were associated with the challenges women faced in accessing the cervical screening they needed. It is well recognized that different immigrant communities, in particular newcomers, will require effective mechanisms of health and health care information, developed with cultural preferences and literacy levels in mind, so that they know how to access primary care services such as cervical screening (Fang & Ragin, 2020). For example, considering the accounts of the women in this study, outreach to Black African immigrant women might be more effectively enhanced by such approaches as having community health nurses reach out via SMS messaging during the BC Pap smear test awareness month than by making fliers or pamphlets available in conventional community locations. Given the findings from these women about communication regarding cervical screening information across generations, it will also be necessary to raise awareness among young girls, including in relation to gender-based stereotypes that may have been passed down from one generation to the next. Such awareness may be essential to ensuring the acceptance of the population based human papillomavirus (HPV) immunization programs designed to prevent cervical cancer in the next generation of women (Litwin et al., 2021). Similarly, information young women obtain about HPV vaccination could open up generational conversations about cervical cancer in general.

All primary care providers, including nurses, should take the initiative to offer cervical screening information and initiate the process at any appropriate point of contact with women of eligible age and be careful not to overly rely on childbearing interactions as the most convenient intervention occasion. At the community and organizational levels, cervical screening awareness might be raised through community outreach programs and awareness-raising workshops in trusted sites such as African worship centers to discourage some of the social norms, cultural, and religious practices that may negatively impact on cervical screening practices and behaviors. Campaigns targeted at wellness centers and African community functions would also likely be worth considering, especially for recent residents who may be overwhelmed by the demands and stress of resettlement and integration in a new country. Finally, as self-administered cervical screening moves beyond the clinical trials phase in Canada (Lofters et al., 2021), it may be appropriate to consider whether this option may be helpful to these women, particularly for those for whom modesty issues are paramount.

Nurses and others interested in developing cervical screening programs for this population might well consider involving community leaders and those from various ethnocultural African groups, and community private organizations, to provide women with opportunities to share their stories and inspire other women. Considering that competing priorities may impact the uptake of cervical screening within this population, as was apparent in the interviews for this study, there may be value in considering methods that have been effective in reaching Black African women in other contexts, through such services as mobile services, such as cervical cancer screening or general women’s mobile health clinics (Malone et al., 2020). This type of geographically accessible program could support community-based options for improving access to information, screening, and service provision across African immigrant women communities.

### Implications for Research

This study has limitations with respect to the number and diversity of the women interviewed, and in some instances, the settings in which they were interviewed because of the complexity of their lives. We also faced the practical reality that in-person interviews and a more ethnographic participant observation approach were not feasible. However, we believe our findings reflect credibility in keeping with the methodological tradition (Thorne, 2016) and offers much to consider as we seek to reduce health inequities within this particular highly vulnerable population subgroup. As social inequality remains a challenge to cancer screening worldwide (Van Hal et al., 2022), and there has been limited research focused on the challenges faced by the sub-Saharan African immigrant population in accessing healthcare in BC, research is currently needed that deeply examines the intersectional effects of social inequality, including what may be considered structural racism (Bailey et al., 2017), on cervical screening uptake in this group. In addition, those interested in supporting this population could build research approaches that integrate advocacy into methodology, working collaboratively with communities to produce social and healthcare systems level change. Considering the current study’s findings, it would seem that inequalities in access to cervical screening and disparities in screening among African immigrant women are both avoidable and unfair in the context of Canada’s universal healthcare system.

## Conclusion

The objective of the comprehensive cervical screening program in British Columbia is to reduce the incidence and number of deaths by increasing the screening rate in the provincial population. Despite developments in cervical cancer screening in Canada, the progress has not benefitted all population groups equitably. Disparities in cervical screening may continue to affect those who are marginalized from the healthcare system and who, through no fault of their own, are faced with complex social challenges. Support for cervical screening among population groups such as Black African immigrant women, therefore, must include a commitment to addressing health disparities through the overall aim of achieving optimal health for all women, paying particular attention to the needs of those who are most at risk for poor health outcomes, including anticipating and ultimately removing socially driven barriers to cancer screening.

## References

[bibr1-23333936241266997] Addawe M. A. Brux Mburu C. Madar A. (2018). Barriers to cervical cancer screening: A qualitative study among Somali women in Oslo Norway. Health and Primary Care, 2(1), 1–5. 10.15761/HPC.1000128

[bibr2-23333936241266997] Adegboyega A. Hatcher J. (2017). Factors influencing pap screening use among African immigrant women. Journal of Transcultural Nursing, 28(5), 479–487. 10.1177/1043659616661612 27470266

[bibr3-23333936241266997] Ali M. A. Ahmad F. Morrow M. (2021). Somali’s perceptions, beliefs and barriers toward breast, cervical and colorectal cancer screening: A socioecological scoping review. International Journal of Migration, Health and Social Care, 17(2), 224–238. 10.1108/IJMHSC-06-2020-0059

[bibr4-23333936241266997] Anaman-Torgbor J. A. King J. Correa-Velez I. (2017). Barriers and facilitators of cervical cancer screening practices among African immigrant women living in Brisbane, Australia. European Journal of Oncology Nursing, 31, 22–29. 10.1016/j.ejon.2017.09.005 29173823

[bibr5-23333936241266997] Bacal V. Blinder H. Momoli F. Wu K. Y. McFaul S. (2019). Is immigrant status associated with cervical cancer screening among women in Canada? Results from a cross-sectional study. Journal of Obstetrics and Gynaecology Canada, 41(6), 824–831.e1. 10.1016/j.jogc.2018.07.010 30361160

[bibr6-23333936241266997] Bailey Z. D. Krieger N. Agénor M. Graves J. Linos N. Bassett M. T. (2017). Structural racism and health inequities in the USA: Evidence and interventions. The Lancet (British Edition), 389(10077), 1453–1463. 10.1016/S0140-6736(17)30569-X 28402827

[bibr7-23333936241266997] Bray F. Ferlay J. Soerjomataram I. Siegel R. L. Torre L. A. Jemal A. (2018). Global cancer statistics 2018: GLOBOCAN estimates of incidence and mortality worldwide for 36 cancers in 185 countries. CA: A Cancer Journal for Clinicians, 68(6), 394–424. 10.3322/caac.21492 30207593

[bibr8-23333936241266997] Caird H. Simkin J. Smith L. Van Niekerk D. Ogilvie G. (2022). The path to eliminating cervical cancer in Canada: Past, present, and future directions. Current Oncology, 29(2), 1117–1122. 10.3390/curroncol29020095 35200594 PMC8870792

[bibr9-23333936241266997] Canadian Cancer Society. (2017). Cervical cancer statistics. http://www.cancer.ca/en/cancer-information/cancer-type/cervical/statistics/?region=on

[bibr10-23333936241266997] Canadian Partnership Against Cancer. (2022). Cervical cancer screening in Canada: 2021/22. https://www.partnershipagainstcancer.ca/topics/cervical-cancer-screening-in-canada-2021-2022/summary/

[bibr11-23333936241266997] Canadian Task Force on Preventive Healthcare. (2018). Summary of recommendations for clinicians and policymakers. https://canadiantaskforce.ca/portfolios/cervical-cancer/

[bibr12-23333936241266997] Carayon P. Wetterneck T. B. Rivera-Rodriguez A. J. Hundt A. S. Hoonakker P. Holden R. Gurses A. P. (2014). Human factors systems approach to healthcare quality and patient safety: Systems ergonomics/human factors. Applied Ergonomics, 45(1), 14–25. 10.1016/j.apergo.2013.04.023 23845724 PMC3795965

[bibr13-23333936241266997] Cénat J. M. Dromer É. Darius W. P. Dalexis R. D. Furyk S. E. Poisson H. Mansoub Bekarkhanechi F. Shah M. Diao D. G. Gedeon A. P. Lebel S. Labelle P. R. (2023). Incidence, factors, and disparities related to cancer among black individuals in Canada: A scoping review. Cancer, 129(3), 335–355. 10.1002/cncr.34551 36436148

[bibr14-23333936241266997] Cha E. Y. Chun H. (2021). Barriers and challenges to cervical cancer screening, follow-up, and prevention measures among Korean immigrant women in Hawaii. Asia-Pacific Journal of Oncology Nursing, 8(2), 132–138. 10.4103/2347-5625.308302 33688561 PMC7934592

[bibr15-23333936241266997] Christy K. Kandasamy S. Majid U. Farrah K. Vanstone M. (2021). Understanding Black women's perspectives and experiences of cervical cancer screening: A systematic review and qualitative meta-synthesis. Journal of Health Care for the Poor and Underserved, 32(4), 1675–1697. 34803036 10.1353/hpu.2021.0159

[bibr16-23333936241266997] DiCicco-Bloom B. Crabtree B. F. (2006). The qualitative research interview. Medical Education, 40(4), 314–321. 10.1111/j.1365-2929.2006.02418.x 16573666

[bibr17-23333936241266997] Fang C. Y. Ragin C. C. (2020). Addressing disparities in cancer screening among U.S. immigrants: Progress and opportunities. Cancer Prevention Research (Philadelphia, Pa.), 13(3), 253–260. 10.1158/1940-6207.CAPR-19-0249 32132119 PMC7080302

[bibr18-23333936241266997] Ferdous M. Lee S. Goopy S. Yang H. Rumana N. Abedin T. Turin T. C. (2018). Barriers to cervical cancer screening faced by immigrant women in Canada: A systematic scoping review. BMC Women’s Health, 18(1), 165. 10.1186/s12905-018-0654-5 30305056 PMC6180489

[bibr19-23333936241266997] Gele A. A. Qureshi S. A. Kour P. Kumar B. Diaz E. (2017). Barriers and facilitators to cervical cancer screening among Pakistani and Somali immigrant women in Oslo: A qualitative study. International Journal of Women’s Health, 9, 487–496. 10.2147/IJWH.S139160 PMC5505544 28740435

[bibr20-23333936241266997] Ghebre R. G. Sewali B. Osman S. Adawe A. Nguyen H. T. Okuyemi K. S. Joseph A. (2015). Cervical cancer: Barriers to screening in the Somali community in Minnesota. Journal of Immigrant & Minority Health, 17(3), 722–728. 10.1007/s10903-014-0080-1 25073605 PMC4312274

[bibr21-23333936241266997] Health Canada. (2019, September 17). Canada’s healthcare system. https://www.canada.ca/en/health-canada/services/health-care-system/reports-publications/health-care-system/canada.html

[bibr22-23333936241266997] Huhmann K. (2020). Barriers and facilitators to breast and cervical cancer screening in Somali immigrant women: An integrative review. Oncology Nursing Forum, 47(2), 177–186. 10.1188/20.ONF.177-186 32078613

[bibr23-23333936241266997] Hulme J. Moravac C. Ahmad F. Cleverly S. Lofters A. Ginsburg O. Dunn S. (2016). “I want to save my life”: Conceptions of cervical and breast cancer screening among urban immigrant women of south Asian and Chinese origin. BMC Public Health, 16(1), 1077. 10.1186/s12889-016-3709-2 27733161 PMC5062908

[bibr24-23333936241266997] Idehen E. E. Pietilä A. Kangasniemi M. (2020). Barriers and facilitators to cervical screening among migrant women of African origin: A qualitative study in Finland. International Journal of Environmental Research and Public Health, 17(20), 1–20. 10.3390/ijerph17207473 PMC7602139 33066565

[bibr25-23333936241266997] Johnson L. G. Armstrong A. Joyce C. M. Teitelman A. M. Buttenheim A. M. (2018). Implementation strategies to improve cervical cancer prevention in sub-Saharan Africa: A systematic review. Implementation Science, 13(1), 28. 10.1186/s13012-018-0718-9 29426344 PMC5807829

[bibr26-23333936241266997] Kilanowski J. F. (2017). Breadth of the socioecological model. Journal of Agromedicine, 22(4), 295–297. 10.1080/1059924X.2017.1358971 28742433

[bibr27-23333936241266997] Litwin C. Smith L. Donken R. Krajden M. van Niekerk D. Naus M. Cook D. Albert A. Ogilvie G. (2021). High-risk HPV prevalence among women undergoing cervical cancer screening: Findings a decade after HPV vaccine implementation in British Columbia, Canada. Vaccine, 39(36), 5198–5204. 10.1016/j.vaccine.2021.07.009 34344555

[bibr28-23333936241266997] Liu C. Wang D. Liu C. Jiang J. Wang X. Chen H. Ju X. Zhang X. (2020). What is the meaning of health literacy? A systematic review and qualitative synthesis. Family Medicine and Community Health, 8(2), e000351. 10.1136/fmch-2020-000351 PMC7239702 32414834

[bibr29-23333936241266997] Lofters A. Devotta K. Prakash V. Vahabi M. (2021). Understanding the acceptability and uptake of HPV self-sampling amongst women under- or never screened for cervical cancer in Toronto (Ontario, Canada): An intervention study protocol. International Journal of Environmental Research and Public Health, 18(17), 9114. 10.3390/ijerph18179114 34501703 PMC8430523

[bibr30-23333936241266997] Malone N. C. Williams M. M. Smith Fawzi M. C. Bennet J. Hill C. Katz J. N. Oriol N. E. (2020). Mobile health clinics in the United States. International Journal for Equity in Health, 19(1), 40. 10.1186/s12939-020-1135-7 32197637 PMC7085168

[bibr31-23333936241266997] Malterud K. Siersma V. D. Guassora A. D. (2016). Sample size in qualitative interview. studies: Guided by information power. Qualitative Health Research, 26(13), 1753–1760. 10.1177/1049732315617444 26613970

[bibr32-23333936241266997] McLeroy K. R. Bibeau D. Steckler A. Glanz K. (1988). An ecological perspective on health promotion programs. Health Education Quarterly, 15(4), 351–377. 10.1177/109019818801500401 3068205

[bibr33-23333936241266997] Ojerinde A. C. (2023). Cervical cancer screening uptake and experiences of Black African immigrant women in the context of a comprehensive provincial screening program in B.C. Canada [Unpublished doctoral dissertation, University of British Columbia]. https://open.library.ubc.ca/soa/cIRcle/collections/ubctheses/24/items/1.0427939

[bibr34-23333936241266997] Okunowo A. A. Daramola E. S. Soibi-Harry A. P. Ezenwankwo F. C. Kuku J. O. Okunade K. S. Anorlu R. I. (2018). Women’s knowledge of cervical cancer and uptake of Pap smear testing and the factors influencing it in a Nigerian tertiary hospital. Journal of Cancer Research and Practice, 5(3), 105–111. 10.1016/j.jcrpr.2018.02.001

[bibr35-23333936241266997] Pierz A. J. Randall T. C. Castle P. E. Adedimeji A. Ingabire C. Kubwimana G. Uwinkindi F. Hagenimana M. Businge L. Musabyimana F. Munyaneza A. Murenzi G. (2020). A scoping review: Facilitators and barriers of cervical cancer screening and early diagnosis of breast cancer in Sub-Saharan African health settings. Gynecologic Oncology Reports, 33, 100605. 10.1016/j.gore.2020.100605 32637528 PMC7327246

[bibr36-23333936241266997] Redwood-Campbell L. Fowler N. Laryea S. Howard M. Kaczorowski J. (2011). “Before you teach me, I cannot know”: Immigrant women’s barriers and enablers with regard to cervical cancer screening among different ethnolinguistic groups in Canada. Canadian Journal of Public Health, 102(3), 230–234. 10.1007/BF03404903 21714325 PMC6975177

[bibr37-23333936241266997] Statistics Canada. (2018). Immigration and ethnocultural diversity in Canada. https://www12.statcan.gc.ca/nhs-enm/2011/as-sa/99-010-x/99-010-x2011001-eng.cfm

[bibr38-23333936241266997] Stokols D. (1996). Translating social ecological theory into guidelines for community health promotion. American Journal of Health Promotion, 10(4), 282–298. 10.4278/0890-1171-10.4.282 10159709

[bibr39-23333936241266997] Stokols D. (2018). Social ecology in the digital age: Solving complex problems in a globalized. world. Academic Press.

[bibr40-23333936241266997] Thorne S. Kirkham S. R. O’Flynn-Magee K. (2004). The analytic challenge in interpretive description. International Journal of Qualitative Methods, 3(1), 1–11. 10.1177/160940690400300101

[bibr41-23333936241266997] Thorne S. E. (2016). Interpretive description: Qualitative research for applied practice (2nd ed.). Routledge.

[bibr42-23333936241266997] Van Hal G. Zeeb H. de Koning H. J. (2022). Editorial: Social inequality in cancer screening. Frontiers in Public Health, 10, 854659. 10.3389/fpubh.2022.854659 35570913 PMC9096238

[bibr43-23333936241266997] WHO. (2023). Cervical cancer-early detection saves lives. https://www.afro.who.int/countries/nigeria/news/cervical-cancer-early-detection-saves-lives#:~:text=In%202020%2C%20Globally%2C%20it%20was,new%20cases%20with%20342%2C000%20deaths.&text=Cervical%20cancer%20ranks%20as%20the,and%2044%20years%20of%20age

[bibr44-23333936241266997] Yimer N. B. Mohammed M. A. Solomon K. Tadese M. Grutzmacher S. Meikena H. K. Alemnew B. Sharew N. T. Habtewold T. D. (2021). Cervical cancer screening uptake in sub-Saharan Africa: A systematic review and meta-analysis. Public Health (London), 195, 105–111. 10.1016/j.puhe.2021.04.014 34082174

